# Values-Based Leadership in Modern Medicine: Lessons From the Naval Submarine Force

**DOI:** 10.7759/cureus.116423

**Published:** 2026-09-17

**Authors:** Alyster A Alcudia, Shacker Mourad, Danielle Heffner, Yoshiya Toyoda, Suyog A Mokashi

**Affiliations:** 1 Division of Cardiovascular Surgery, Lewis Katz School of Medicine, Philadelphia, USA; 2 The Wharton School, University of Pennsylvania, Philadelphia, USA; 3 Division of Cardiovascular Surgery, Temple University Hospital, Philadelphia, USA

**Keywords:** high-reliability organizations, just culture, medical errors, patient safety, psychological safety, quality improvement, surgical sabermetrics, values-based leadership

## Abstract

Preventable medical errors remain a leading cause of morbidity and mortality in the United States. While healthcare systems have achieved safety improvements by adopting protocols from commercial aviation, such as surgical checklists and Crew Resource Management, broad safety gains have arguably stalled. Commercial aviation relies on highly standardized environments, whereas healthcare is characterized by extreme biologic unpredictability and the need for rapid, decentralized decision-making. To break through this safety plateau, healthcare leaders must dismantle rigid hierarchies, intimidating behaviors, and individualistic clinical command archetypes. This review posits that the cultural transformation achieved by the U.S. submarine force and the Naval Nuclear Propulsion Program provides a highly analogous and necessary blueprint for modern healthcare leadership. By mapping naval high-reliability principles across six operational domains of organizational leadership, i.e., Leading with Values, Talent Management, Setting a Clear Direction, Effective Communication, Motivation and Team Engagement, and Execution, this review demonstrates how healthcare professionals can cultivate resilient, high-trust cultures. It also highlights the necessity of adopting a “Just Culture” to prioritize systems learning over individual blame. It emphasizes selecting and developing talent based on character, establishing shared mental models via Commander’s Intent, and flattening authority gradients through structured communication and approachability training. Furthermore, the framework underscores the importance of cultivating psychological safety and clinical empathy to motivate teams, alongside maintaining zero-defect execution through standardized checkpoints, closed-loop administrative pathways, and emerging technologies like surgical sabermetrics. Ultimately, values-based leadership transcends compliance-driven management by treating leadership as an active, daily practice rather than a positional title. By bridging the gap between individual technical skills and systemic organizational resilience, this philosophy provides a robust operating system for health systems to eliminate preventable harm and uphold the highest standards of patient-centered care.

## Introduction and background

More than two decades after early landmark reports on patient safety, preventable medical errors remain a leading cause of morbidity and mortality (M&M) in the United States [1]. In response, healthcare systems have aggressively pursued quality improvement initiatives, largely by adopting safety protocols from commercial aviation, such as surgical checklists and Crew Resource Management (CRM) [1]. While these interventions have yielded significant improvements, broad healthcare safety gains have arguably stalled, with preventable harm remaining stubbornly common [2]. Commercial aviation relies on highly standardized and predictable environments; conversely, healthcare is characterized by biologic unpredictability, extreme complexity, and the need for rapid, decentralized decision-making [3]. As Chassin and Loeb [4] argue, incremental policy additions and one-size-fits-all mandates require a genuine culture of safety to produce high reliability, which is defined as an organization’s capacity to operate under hazardous, complex conditions while sustaining exemplary safety through collective mindfulness and resilience. Achieving such a safety culture, however, requires dismantling the rigid hierarchies, intimidating behaviors, and individualistic archetypes of clinical command that persist across many healthcare environments [5].

To break through this plateau, healthcare leaders must look beyond commercial aviation to a more analogous model of high reliability: the Naval Nuclear Propulsion Program and the U.S. submarine force. A fast-attack submarine and an operating room are both isolated, high-pressure micro-environments where highly trained, multidisciplinary teams operate under zero-margin-for-error constraints. When a catastrophic anomaly occurs, the team must rely entirely on their shared mental models, communication protocols, and mutual trust to survive. This review posits that the cultural transformation achieved by the naval submarine force provides a desperately needed blueprint for modern healthcare leadership.

However, it is critical to note that the organizational deployment of values-based leadership cannot succeed without a foundational commitment by individual leaders. As previously discussed by Mokashi [6], clinical leaders must first master Kraemer’s four personal principles of self-reflection, balance, true self-confidence, and genuine humility to successfully navigate the tension between administrative constraints and clinical autonomy. This internal mastery serves as the prerequisite for executing external, organizational strategy, shifting the organizational focus from superficial hospitality metrics toward meaningful functional outcomes and patient safety [7].

Building on that foundation, this review maps naval high-reliability principles across Kraemer’s six operational domains of organizational leadership: Leading with Values, Talent Management, Setting a Clear Direction, Effective Communication, Motivation and Team Engagement, and Execution (Table 1). By doing so, this review demonstrates how healthcare professionals can move beyond compliance-driven management to cultivate the distributed leadership, active followership, and high-trust cultures necessary to eliminate preventable harm [5].

**Table 1:** Applying Kraemer’s values-based domains to healthcare high reliability. M&M: morbidity and mortality; NOTSS: Non-Technical Skills for Surgeons; SBAR: Situation, Background, Assessment, Recommendation.

Leadership domain	Healthcare and high-reliability organization (HRO) application	Desired outcome
Leading with Values	Utilizing “Just Culture” frameworks and structured M&M analyses to address cognitive/systemic errors	Systemic learning over individual blame; ethical transparency
Talent Management	Integrating non-technical skills (NOTSS) and formal communication training into early career pipelines	High retention of character-driven staff; sustained reductions in clinician burnout
Setting Direction	Utilizing Commander’s Intent to establish shared mental models and align goals around patient outcomes	Frontline autonomy; explicit role clarity during complex clinical procedures
Effective Communication	Flattening authority gradients through approachability training, SBAR, and closed-loop communication	Elimination of assumption traps; uninhibited flow of safety information across all ranks
Team Engagement	Cultivating a “questioning attitude,” cognitive empathy, and TeamSTEPPS to dismantle autocratic command	High psychological safety, resilient therapeutic alliances, and interdisciplinary trust
Execution	Implementing administrative effector pathways, the SURPASS checklist, and surgical sabermetrics to monitor cognitive load	Zero-defect execution; continuous translation of clinical feedback into systemic policy remediation

Methods: literature search strategy

An integrative literature search was conducted across PubMed (National Library of Medicine, Bethesda, Maryland), Google Scholar (Google LLC, Mountain View, California), and EBSCOhost (EBSCO Information Services, Ipswich, Massachusetts) (including CINAHL and Business Source Ultimate) to identify peer-reviewed literature bridging high-reliability organizational (HRO) principles, military/naval command cultures, and clinical patient safety. Searches were conducted between July 15, 2026, and August 15, 2026, including studies published from January 1, 2000, through July 1, 2026.

Iterative searches were applied to titles and abstracts using primary search terms, and conceptual combinations included the following: (“values-based leadership” OR “supportive leadership” OR “intent-based leadership”) AND (“high-reliability organizations” OR “nuclear submarine” OR “naval nuclear propulsion”) AND (“patient safety” OR “Just Culture” OR “psychological safety” OR “medical errors” OR “SBAR”).

Eligible studies included English-language, peer-reviewed original investigations, systematic and narrative reviews, and organizational case analyses addressing cross-industry safety adaptations, authority gradients, and leadership development. Editorials, popular press articles, and non-peer-reviewed magazine or NGO commentaries were excluded to maintain academic rigor.

Due to the cross-disciplinary nature of this review, literature was purposively sampled to bridge naval operational frameworks with healthcare safety science. Literature was iteratively retrieved and selected based on conceptual relevance, with final selections reviewed and refined in collaboration with senior leadership. Because this review synthesizes cross-disciplinary leadership paradigms rather than evaluating specific clinical interventions, formal clinical risk-of-bias tools were not applicable. Instead, source quality was evaluated qualitatively based on academic rigor, historical authenticity, and direct applicability to the leadership and patient safety concepts discussed. The final selection of literature represents an integrative synthesis of these intersecting fields.

## Review

Leading with values: just culture and systems learning

In the U.S. Navy, the tragic loss of the USS Thresher in 1963 was not viewed as an isolated mechanical breakdown but rather as a consequence of rapid technological complexity outstripping Industrial Age quality control and institutional discipline [8]. This crucible moment gave rise to the Navy’s rigorous Submarine Safety (SUBSAFE) certification program, a comprehensive quality assurance and material control framework governing submarine design, construction, and maintenance to guarantee hull and flooding integrity under deep submergence [8]. Concurrently, the disaster enabled Admiral Hyman G. Rickover to institutionalize an uncompromising ethical culture of engineering discipline, technical competence, and strict procedural adherence across the naval nuclear enterprise [8].

Healthcare must mirror this commitment through the adoption of a “Just Culture,” an environment of balanced accountability where individual actions and systemic design are evaluated fairly [9]. Traditional medical hierarchies have historically relied on a person-based approach to human error, which fosters a culture of individual blame and suppresses transparent reporting [1]. This punitive model drives worldwide underreporting out of fear, effectively blinding organizations to the latent system failures that cause recurring medical errors [10]. As Mountford and Lee [5] argue, transcending this archetype requires moving away from individualistic, heroic notions of clinical command toward cultures of distributed leadership and complex healthcare systems. In complex healthcare systems, values-based leaders recognize that psychological safety is maintained not only when subordinates are told to “speak up” but also when leaders actively “listen up” and design processes that prioritize orderly learning over punitive posturing [5].

Values-based healthcare leaders, however, approach errors as systems-design problems. The M&M conference serves as a primary vehicle for this. Transforming M&Ms from adversarial, shame-based rituals into structured engines of institutional learning requires analyzing cognitive biases and identifying latent systems vulnerabilities [11]. By standardizing error taxonomies and embedding high-reliability principles, such as a “preoccupation with failure” and a “reluctance to simplify,” leaders can prioritize systemic remediation over individual culpability, aligning clinical governance with enduring ethical values [12].

Talent management: selecting and developing resilient teams

Long-term organizational success hinges on selecting and developing individuals for their intrinsic character, not just their technical prowess. During World War II, Captain Eugene Fluckey transformed the USS Barb into a highly effective submarine by deploying talent based on rigorous psychological selection, continuous cross-training, and a philosophy of driving oneself while empowering the crew [13].

In modern medicine, technical dexterity and non-technical interpersonal skills are similarly interdependent. Integrating frameworks like the Non-Technical Skills for Surgeons (NOTSS) alongside technical training has been shown to enhance systemic operational resilience and increase psychological safety during unexpected surgical crises [14]. However, structural pipelines for leadership remain inadequate. Recent studies show that while 71.5% of senior nursing students have held leadership positions, only 20% received formal leadership training, leaving them to struggle with heavy academic workloads and fears of peer judgment [15]. Similarly, current healthcare executives report that relational leadership and systems-based thinking are the most critical skills they wish they had developed earlier in their careers [16].

Addressing this deficit requires redefining medical professionalism itself. Healthcare has historically celebrated an individualistic archetype of command, often tolerating disruptive, autocratic behaviors so long as technical skill is high [5]. Values-based organizations must dismantle this model by framing leadership not as an elite positional honor but as a daily practice cultivated from the earliest stages of training. Concurrently, there must be a deliberate cultivation of followership, with team members being developed to actively support ethical leaders, check emerging errors, and serve as an organizational defense against toxic authority [5].

Finally, talent management requires protecting and retaining the workforce. Structured relational training pays compounding dividends in clinician well-being: large-scale health system interventions demonstrate that formal communication and interpersonal skills training significantly increase clinical empathy while producing sustained reductions in physician burnout and depersonalization [17]. By treating relational leadership, active followership, and emotional resilience as core clinical competencies rather than optional auxiliary skills, healthcare organizations can build an enduring talent pipeline capable of navigating high-hazard environments.

Setting a clear direction: shared mental models and commander’s intent

In environments characterized by inherent complexity, leaders must distill intricate information into a simple, shared mental model. The military achieves this through Commander’s Intent, an operational philosophy that empowers subordinates to act autonomously by ensuring they deeply understand the overarching goal of a mission. Captain L. David Marquet explored and formalized this concept into Intent-Based Leadership (IBL) during his command of the USS Santa Fe [18]. Moving away from an authoritarian “leader-follower” structure to an IBL-driven “leader-leader” paradigm fosters psychological ownership and activates frontline critical thinking [19]. This philosophy is sustained today by operational models that reject passive followership in favor of a “leader-leader” paradigm, explicitly prioritizing decentralized execution and immediate action during high-stakes crises [20].

In healthcare, setting a clear direction begins with defining the organization’s overarching “North Star.” As Merlino [7] emphasizes, true patient-centered care has evolved beyond superficial customer-service initiatives or hospitality metrics of patient “happiness.” Instead, values-based clinical direction must be anchored in what patients value most: delivering appropriate, seamless care across the continuum that maximizes measurable functional outcomes and eliminates preventable harm [7].

However, establishing a broad strategic mission is futile if it fails to translate into frontline operational clarity. Mobilizing healthcare teams requires operationalizing three foundational leadership responsibilities originally articulated by industrial leader Paul O’Neill: ensuring every team member is clear about their specific role, making them understand how that role fits into the larger collective purpose, and equipping them with the tools and respect needed for execution [5].

When leaders fail to establish this clarity, shared mental models fail. A prospective study of multidisciplinary operating room teams demonstrated that while clinicians largely agreed on the chronological sequence of surgical tasks (averaging 87% similarity), agreement regarding who was responsible for executing those tasks averaged only 70% and collapsed to just 51% for critical patient safety checkpoints such as estimating blood loss, verifying warming devices, and confirming patient positioning [21]. Ambiguous role expectations create dangerous operational vulnerabilities where vital safety tasks are either duplicated or omitted entirely. Healthcare leaders must explicitly calibrate both the overarching mission and specific roles and responsibilities prior to high-risk clinical interventions, ensuring the entire team operates from a synchronized mental model before undertaking complex procedures.

Effective communication: flattening the authority gradient

Effective communication is a continuous, transparent process that relies on both structured protocols and the elimination of steep authority gradients. On a nuclear submarine, complex operations are managed through strict procedural discipline, utilizing high-bandwidth, closed-loop reporting standards to eliminate assumption traps. This procedural formality is not intended to be oppressive; rather, empirical analyses of submarine crews demonstrate that structured communication turn-taking serves as the foundational discipline that holds team cohesion together, ensuring operators instinctively revert to safe patterns when overwhelmed by stress and complexity [22,23].

In healthcare, the need for similar communication discipline is acute. Communication failures are the root cause of approximately 52% of surgical flow disruptions and contribute to 65% of Joint Commission sentinel events [24]. Implementing structured communication tools, such as the Situation, Background, Assessment, Recommendation (SBAR) framework and formalized surgical timeouts, mirrors naval formality and has been shown to significantly reduce preventable harm in the operating room [24].

However, structured tools alone are insufficient if team members feel too intimidated to use them. Healthcare must actively flatten the authority gradient that has historically dominated clinical hierarchies. Empowering junior staff through “approachability training” ensures that vital safety information flows freely across all ranks, establishing an environment where any team member can voice concerns without fear of humiliation or retaliation [1]. Looking forward, healthcare can further bolster these efforts by integrating artificial intelligence (AI) and large language models (LLMs) to monitor clinical communication in real time, flagging missed closed-loop checks and serving as virtual coaches during debriefs, thereby evolving CRM for the digital age [1].

Motivation and team engagement: psychological safety and the questioning attitude

The U.S. submarine force maintains its operational edge through an institutionalized culture of rigorous self-assessment and a relentless “questioning attitude.” Stemming from the uncompromising standards of Admiral Rickover, submariners are expected to challenge assumptions, ask questions, and communicate hard truths to superiors without fear of hierarchical reprisal [13,25].

In healthcare, achieving this level of operational vigilance requires leaders to actively cultivate psychological safety. Notably, psychological safety is not a static organizational trait but a fragile state; recent longitudinal data reveal that while healthcare professionals often arrive with high psychological safety, it rapidly atrophies without active maintenance from departmental leadership [26]. In high-risk clinical environments, technical brilliance is frequently undermined when leaders suppress a questioning attitude through intimidation or indifference. A baseline audit of a cardiothoracic operating room revealed that only one-third of surgeons met acceptable emotional intelligence cutoff values, correlating directly with suppressed psychological safety (where approval hovered between 35% and 50%), high workforce turnover intentions, and a profound reluctance to speak up about safety hazards [27]. To counteract this silence, structured team training programs like TeamSTEPPS explicitly teach mutual support, shared mental models, and conflict resolution, thereby mitigating burnout and restoring team engagement [27].

The style of leadership is equally critical to team motivation. Fukami [3] contrasts traditional authoritarian “Boss-style” command (“Do it!”) with collaborative “Captain-style” engagement (“Let’s Go!”) and humble “Follower-style” enablement (“How’s it going?”). Supportive leaders recognize that high reliability cannot be enforced through top-down mandates; it requires cultivating an environment where team members feel psychologically safe to report errors and innovate without fear of punitive consequences [3].

A foundational element of this supportive environment is clinical empathy. Often mischaracterized as mere soft sentimentality, clinical empathy is actually a structured, cognitive discipline that builds profound relational trust [28]. When leaders and clinicians practice active empathy, they not only strengthen the therapeutic alliance, leading to improved objective clinical outcomes like better metabolic control in diabetic patients, but also provide a crucial buffer against systemic burnout, keeping the healthcare team resilient, fulfilled, and engaged [28]. Crucially, empathy and relational engagement can be systematically trained: large-scale health system data demonstrate that formal, experiential communication curricula yield statistically significant increases in physician empathy scores while producing sustained reductions in depersonalization and burnout [17]. By prioritizing supportive leadership, psychological safety, and cognitive empathy, healthcare organizations motivate resilient teams capable of maintaining constant vigilance under zero-margin constraints.

Execution and implementation: flawless execution under zero-margin constraints

Flawless execution in zero-margin environments is the ultimate test of a team’s planning, feedback loops, and shared values. Within Naval Nuclear Propulsion, safe reactor operation relies on a symbiotic relationship between rigorous, top-down operating procedures and the bottom-up vigilance of junior watchstanders. This dynamic reflects the balance between “organizational mindfulness,” i.e., the enduring, top-down structures and practices established by senior leaders, and “mindful organizing,” which is the fragile, ongoing social process of real-time communication enacted by frontline workers [29]. Middle managers serve as the crucial bridge between these executive strategies and actionable frontline routines.

To achieve a comparable maturity model in healthcare, hospital leadership must fundamentally commit to the ultimate goal of zero patient harm [4]. Standardized verification checkpoints, such as the comprehensive SURPASS checklist, provide a vital operational framework; when executed with procedural formality across the entire surgical pathway, this system has been shown to significantly reduce postoperative complications and in-hospital mortality [30].

Achieving zero-defect execution also requires aligning operational measurement with what truly matters. As Merlino [7] emphasizes, healthcare systems cannot evaluate execution through superficial customer-service metrics or patient amenities; high-reliability organizations align their operational compass around measurable patient-reported outcome measures (PROMs), scalable acute-care delivery innovations, and value-based incentives that hold systems accountable for functional recovery and zero harm across the longitudinal care continuum [7].

However, simply identifying errors through checklists or M&M conferences is insufficient without a closed-loop administrative apparatus. M&M rounds must be integrated with explicit administrative effector pathways that assign accountability, generate specific action plans, and track the implementation of quality improvement initiatives [31]. For example, the implementation of a structured M&M model with dedicated quality committees at one tertiary care center resulted in 45 distinct hospital policy changes, demonstrating the power of formal mechanisms to translate discussion into systemic remediation [32].

Furthermore, true operational reliability requires the continual, objective measurement of human performance. Validated behavioral marker systems, such as the NOTSS tool, are essential for systematically evaluating and cultivating cognitive and interpersonal competencies like situational awareness and task management [14,33]. Emerging technologies such as “surgical sabermetrics” elevate this by utilizing non-invasive physiological sensors to digitally assess these non-technical skills in real time [34]. By objectively measuring a surgeon’s Cognitive Load (CogL) through autonomic nervous system responses, such as heart rate variability or electroencephalography, these tools can identify and prevent the cognitive saturation that precedes human error, optimizing operative performance and safeguarding patient safety [34].

Limitations

This review has several limitations that warrant consideration. First, fundamental structural differences exist among the operational domains synthesized. While nuclear submarines operate under a unified military chain of command with centrally mandated procedural compliance [8], and Kraemer's leadership principles stem from corporate executive management [9], healthcare organizations are characterized by decentralized authority, multiple competing professional hierarchies, and extreme biological unpredictability [4,5]. High-reliability practices cannot be ported directly without accounting for these distinct governance structures. Second, an inherent selection bias exists within the search methodology: while health sciences literature is indexed comprehensively in academic databases like PubMed (National Library of Medicine, Bethesda, Maryland) and CINAHL, foundational naval operational literature frequently resides in professional military periodicals, declassified government inquiry records, and service-branch monographs that fall outside standard peer-reviewed indexing [8].

## Conclusions

The contemporary healthcare environment demands a leadership approach that transcends traditional, compliance-driven management and embraces the inherent complexities of human interaction, ethical decision-making, and dynamic operational challenges. As this exegesis of Harry Kraemer’s framework demonstrates through the lens of Naval Nuclear Propulsion and high-reliability science, values-based leadership provides a robust and adaptable operating system for modern health systems.

These principles of encompassing the demonstration of values via Just Culture, strategic talent management, the clarity of direction anchored in functional outcomes, the power of structured communication, the cultivation of psychological safety and active followership, and the discipline of zero-defect execution are not isolated concepts. Instead, they are interconnected components of a holistic leadership philosophy. When consistently applied, these competencies empower leaders across all healthcare domains to dismantle toxic hierarchies, foster resilient interprofessional teams, drive meaningful innovation, and uphold the highest standards of ethical patient care. Ultimately, achieving high reliability requires recognizing that leadership is an active daily practice rather than an elite positional title. By bridging the gap between individual technical skills and systemic organizational resilience, values-based leadership enables healthcare systems to achieve the true triple aim of improving the personal experience of care, advancing population health, and reducing per capita costs. Embracing and developing these values-based competencies is not just advantageous; it is an enduring imperative for all who aspire to lead effectively in the vital field of healthcare.
